# Demand for Space: Elderly Transgender and Gender Nonconforming People, Healthcare, and Theological Ethics

**DOI:** 10.1007/s10943-020-01101-9

**Published:** 2020-10-26

**Authors:** Mathias Wirth

**Affiliations:** Department of Systematic Theology/Ethics, Faculty of Theology, University of Bern, Längassstrasse 51, 3012 Bern, Switzerland

**Keywords:** Elderly, Gender and ethics, Gender and religion, Healthcare ethics, Theological ethics and space, Theological ethics and withdrawal, Trans health

## Abstract

Visibility for transgender and gender nonconforming people and the elderly is growing; however, thus far the overlap of the two groups has rarely been considered. Trans persons therefore remain largely invisible in the context of older people’s care and medicine. The discrimination faced by this group is at least twofold: they are the targets of aggression incited by transphobia, and also by ageism. Although older trans and gender nonconforming people exist as a greatly marginalized group within another already marginalized group, even the field of theological ethics has neglected to grant them ethical attention. This leads to especially harsh consequences for elderly transgender and gender nonconforming people due to their specific vulnerabilities. There are reports from trans persons who have resolved never to make use of health services again due to regular experiences of transphobia in medical settings. There are religious components within transgender and gender nonconforming issues that should not be overlooked in this context. On the one hand, medical staff, in the name of their Christian beliefs, have refused to provide trans persons with basic medical care. On the other hand, demands for places of visibility, and spaces for the individual, are regularly made in trans-positive studies, and can be linked to discussions within theological ethics about giving space. Some ethical formulas within the Hebrew and Christian traditions focus on the creation of space in which other beings may exist, as found in concepts like brother–sisterhood, friendship, and Sabbath. By casting light on elderly trans and gender nonconforming people, and on their demands for space, via reflections on ethical concepts of space-making, this study develops a specific understanding of space for elderly trans persons. The paper aims to develop an understanding of trans-positive spaces within theological ethics and applied ethics. Spaces that assume a withdrawal or contraction by all those who have previously taken up trans spaces through ignorance, contempt, or violence, should not thereby become spaces of absence: indeed, elderly trans and gender nonconforming people might be in need of both kinds of spaces, those where otherness enables withdrawal, and those where the helping presence of others continues.

Visibility for transgender and gender nonconforming people and the elderly is growing (Singh and Bower 2018); however, thus far, the overlap of the two groups has rarely been considered (Appenroth and Lottmann 2019; Cook-Daniels 2006; Witten 2014). Scarcely any research has been done on the medical and psychological treatment of elderly trans persons (Dragon et al. 2017; Fabbre 2015); in fact, older trans individuals are one of the most underrepresented groups within medical research (Carroll 2017). It has not yet been acknowledged in medical-ethical debate that the discrimination faced by this group is threefold: even in a clinical setting (Blevins and Werth 2006; Yarbrough 2018), they are the target of aggression incited by transphobia due to their gender identity (Beemyn and Rankin 2011), by homophobia due to their possible sexual orientation, and by ageism due to being old (Kimmel et al. 2006). Thus, although older trans persons exist as a greatly marginalized group within another already marginalized group (Arthur 2015; Shelley 2008; Singh and Bower 2018), even the field of theological ethics has neglected to grant them ethical attention, despite data regularly being collected on the places and spaces of visibility and on dignity in the areas of gender and age.^1^ Theological ethics can build on this.

Even before factoring in elderliness, one of the hurdles trans and gender nonconforming people encounter is finding medical practitioners who both support genuine trans medical interventions, such as sex reassignment surgery, and also guarantee general medical care. They face an often-difficult search for trans-friendly medical facilities where trans persons are not subjected to discrimination or divesting behavior. An overwhelming majority of trans persons claim to have had experiences in medical settings that range from discriminatory to traumatizing (Shelley 2008). This experience is valid, at least for the Western context this study focusses on, with regard to literature from both English-speaking (USA, UK) and German-speaking countries (Germany, Switzerland). The perspective of no longer being able to care for oneself in old age, and being cared for by staff with no sensitivity toward or knowledge of transgender issues, proves to be one of the greatest worries for the future reported by trans persons (Transgender Network Switzerland 2018): “I shudder to think what’s going to happen when I am less able to self advocate, I am afraid of there not being trans-spaces as an elder, spaces where our bodies are being handled and moved around and manipulated for basic functioning. […] I just don’t know, and it really scares me” (Anonymous, quoted in Witten 2014, p. 26).

## Transgender: Concept, Medicine, Ethics

The transgender phenomenon is by no means new; time and time again, people have liberated themselves from the format of assigned gender in very different eras and cultural contexts, even if only temporarily (Wirth 2015, 2019). However, a new type of trans visibility is emerging (Beemyn and Rankin 2011). One example of this development is the trans themed week to which the broadcaster Swiss Radio and Television (SRF) dedicated several broadcasting formats in Spring 2018. For the context of this study, it is interesting that this initiated a round of talks in the broadcasting series “Decisive Moments in Religion” (German: *“Sternstunden Religion”*). Obviously, religious, theological, and ethical issues also feature in the transgender debate for a wider audience.^2^ Such theological and ethical references will be returned to later.

What is understood as transgender or, more broadly, as trans can indeed be learned about in academic debates in the fields of medicine, psychology, law, and in particular the specialized cultural science Gender Studies. But first and foremost, this is learnt from trans persons themselves. Their definitions and classifications, such as those formulated by *Transgender Network Switzerland* (TGNS), serve here not only as an introduction, but as a reference point.

Terminologically, the term “transgender” is the most common and widely accepted. It is a term also used by the TGNS, though the broader term “trans” is preferred. Trans describes the state in which a person experiences dissonance between their gender identity and the gender they were assigned at birth. By extension, according to the TGNS, which here is representative of the trans-internal perspective, for transgender people it is not about the desire to swap genders, but quite the opposite, instead aligning their gender expression with their gender identity (Transgender Network Switzerland 2018). This is exactly how it is described by Alex, who was designated female at birth, but identifies as male: “I always just felt wrong, like something wasn’t right and I hadn’t a clue as to what that was. […] Nobody talks about this stuff because they don’t want it to exist. Especially the fundamentalists out there. And it’s not a choice. Not many people feel like they are in the wrong body for the fun of it” (cited in Girshick 2008, p. 154).

Although some trans persons identify with both genders (bigender) (Beemyn and Rankin 2011), the TGNS’s definition applies to most trans persons as they do not identify with their assigned gender (anymore). However, other writers who indicate the potential for “motion, movement, process” inherent in the “trans” prefix as characteristic of transgender people (Wirth 2018) therefore defy labels but emphasize the bitter commonality that all trans persons would share in being victims of transphobia (Shelley 2008). Furthermore, in the interests of the queer paradigm, others change nomenclatures, definitions, and designations—like the 27-year-old Ivan: “Being queer is for me a sociopolitical stance. Sometimes I describe my gender identity as trans, sometimes as queer, and sometimes just as male. I am simply myself, Ivan” (Transgender Network Switzerland 2018, p. 8).^3^

Being transgender or trans or gender nonconforming is different than being lesbian, gay, or bisexual; that is to say, it is not about sexual orientation. The sexual preferences of trans persons are no different than those of cisgender individuals.^4^ In this way, it is not unusual if a trans woman born with male genitals feels romantically and sexually attracted to women, living in a lesbian relationship after gender reassignment, which prior to the transition would have been defined as heterosexual. For transgender people, the greater focus is on gender identity^5^ in the cosmos of sexuality, in which male and female exist as two predominant but not sole variants. It could be said that sexual orientation is primarily concerned with the “where to” of sexuality and gender, and that transgender people are an important example of gender orientation, which is primarily concerned with the “where from” of gender and, consequently, sexuality.

In practical terms, gender reassignment, understood as the desire for medical medicinal and surgical interventions (Adam’s apple shaving, breast augmentation or mastectomy, vocal fold shortening, phalloplasty, etc.) is often a life-long journey. In particular, hormones, which can come in the form of tablets, gels, patches, or injections, must in most cases be taken continually (Transgender Network Switzerland 2018). Depending on jurisdictions, a psychological assessment forms part of gender reassignment, often related to the task of confirming the authenticity of trans feelings before reassignment therapies are allowed or are covered by insurance.^6^ This often leads to an initial mistrust between trans persons and therapeutic staff (Davy 2010) who appear as gatekeepers (Gherovici 2010).^7^ The long history of medicine and in particular psychiatry in Western cultures must not be forgotten: still to this day, it emerges as a representative of normality rather than a representative of the individual in appearance, pushing trans persons toward integration (Shelley 2008).^8^ Meanwhile, proof increasingly suggests that reassignment therapies improve the mental condition of trans persons. With a focus on transgender adults, studies show fewer tendencies toward suicide, substance abuse, and isolation in comparison to peers who are not undergoing reassignment therapies (Beckwith et al. 2017; Tinney et al. 2015).

The general medical care of trans persons is problematic (Beemyn and Rankin 2011; Yarbrough 2018) due to barriers (fear of discrimination, few specialists, financial concerns, etc.) and continued failing standards of care in all four countries this study is focusing on (i.e. Germany, Switzerland, UK, USA).^9^ There are authors who stress that the institutional exclusion of trans persons can most clearly be seen in the medical sector. There are reports from trans persons who have resolved to never again make use of health services due to regular experiences of transphobia in medical settings, completely refusing medical treatment and often-necessary life-long check-ups that are particularly important when on hormone therapy and for the early detection of (specific) types of cancer (Arthur 2015; Shelley 2008). Trans people often decide against even acute care (Gangamma 2008). On the whole, therefore, older trans persons are at huge risk from poor physical and mental health (Shelley 2008), only made worse by the fact that it is so difficult to find practitioners, such as gynecologists and psychiatrists, who provide treatment for trans persons (Shelley 2008).^10^ In the context of the USA, it can be assumed that the average trans person is in poor health (Beemyn and Rankin 2011). This is made even more serious by the especially high incidence rate of mental and even physical injuries in trans persons who would require comprehensive medical and therapeutic care (Shelley 2008). Nevertheless, there are also positive developments taking place under the rubric of “Transgender Health” that strive for increased “Transgender Awareness” in the field of healthcare (Hanssmann 2012). One example thereof includes the training of medical staff on the needs of trans persons (Hanssmann 2012) which has already been offered in pilot projects in medical study programs in the US (Park and Safer 2018).

Furthermore, there are religious components within transgender and gender nonconforming issues that should not be overlooked. Trans persons experience animosity in the context of Western Christianity (Beemyn and Rankin 2011), especially due to the now-secularized concept of the dichotomy of the sexes in Genesis 1:27. This is also reflected in internalized forms of transphobia where trans persons tend to harbor negative feelings toward their sense of gender for religious reasons (Girshick 2008; Fabbre 2015). Trans persons with a Christian background report that all of their shame was or is outlined by religion (Girshick 2008; Fabbre 2015); however, there are cases to the contrary where trans persons’ faith has inspired them with strength: “I never believed I would go to hell just for being the person God made me” (Girshick 2008, p. 172). If trans people appear to use religious and spiritual references, then the danger exists that religious affiliations with its supportive meaning (Noth 2013) can also cause grief and stress (Arthur 2015; Blevins and Werth 2006; Porter and Krinsky 2014). Medical staff, in the name of their Christian beliefs, have even refused to provide trans persons with basic medical care, trans persons from English-speaking countries have reported.^11^ Complex are the causes of the rejection of trans persons, which is by no means limited to the medical sector, although it is particularly severe since it regularly prevents trans persons from seeking medical help. Alongside the focus on the (male) genitals in Christian culture (Wirth 2015), changing this part of the body is treated differently than, for example, surgery on the lips or ears because trans persons invalidate the dogma of gender binarism (Davy 2010). In this way, however, they shatter a seemingly fundamental structural order (Beemyn and Rankin 2011). This seems to be linked to the Genesis formula of “male and female he created them” (Gen 1:27), since this line is also cited in completely non-theological analyses of transness as a powerful *(wirkungsmächtiges)* concept (Gherovici 2010), even when it is shown that the story of creation should not be interpreted as a rejection of the possible existence of trans persons.^12^ In any case, the diffusion of specific Christian images and rhetoric (Jaarsma 2009), and the way that this shapes experiences of reality (Fischer 2004), means theology has a lasting responsibility for the interpretation of its “export hits”. A latent loyalty toward Christianity or even a certain rarer sense of religious duty in some Western cultures is, of course, expressed precisely at the place of gender (Shelley 2008).

## Older Age: Concept, Medicine, Ethics

Similarly to being trans, the concept of older age is ambiguous and refers to a variety of possible states (Bozzaro 2014). Across the different manifestations of older age it appears that older people have already lived out most of their lives. So as not to succumb to despair, this fact can be pushed to the back of one’s mind, for example, or a special significance can be given to the time remaining. Thus, personal goals and the belief in one’s own possibilities also continue to play a central role in this stage of life (Elsässer et al. 2017). This manifests itself not only in goal engagement but also in goal disengagement (Elsässer et al. 2017). This is necessary because with older age comes the greater likelihood of falling ill, which often results in limitations. Common diseases such as diabetes, cancer, and cardiovascular disease become more frequent. Furthermore, there is an increase in chronic inflammation (for example in the joints and the blood vessels). Diseases of the brain and the nervous system are also associated with older age. Nonetheless, equating aging to only physical, mental, and social deterioration (Schweda et al. 2018) would be unreasonable. First, it is not written in stone that specific illnesses will inevitably occur at specific ages (Elsässer et al. 2017). Second, as indicated by psychological research into aging (gerontology) in particular, it also leads to age-specific skills (for instance, crystallized intelligence such as literacy) and to the accumulation of certain competences (for instance, knowledge on history, norms, particular relations, social behavior, etc.) (Elsässer et al. 2017). Interdisciplinary research into aging highlights the plasticity and malleability of age that is associated with high individuation and a wide range of variants (Coors 2014; Schweda et al. 2018) and whose perception, similarly to that of gender, is shaped by cultural standards, not just by biological factors (Zissler 2016).

Nonetheless, old age remains an especially vulnerable phase of life (Coors 2020; Schweda et al. 2018; Werren 2019). This is related to widespread negative stereotypes based on age as prevalent in contemporary Western countries (Elsässer et al. 2017). Attitudes toward old age vary more than those toward any other stages of life: in some epochs it is considered glorious, whereas in others it is viewed with pure contempt (Knell 2017). In any case, no other life stage is subject to so many not only clear but also divergent judgements. Presently, the negative stereotypes of old age form the majority. An “ageing society” (Vierck and Hodges 2003, p. 4) is not associated with maturity, but rather with burdening the social security system. From this perspective older people, particularly the very elderly, only seem to be a burden (Knell 2017). A negative view of older people and a disregard of their interests is called *ageism*. It exists alongside racism and sexism and describes a particular form of discrimination (Knell 2017; Zissler 2016).^13^ Most notably, it means treating age as a particularly significant factor—just as with gender, incidentally. This then leads to generalized assumptions being made about a person (Kimmel et al. 2006). In a medical context, ageism most commonly finds expression in considering (and in some places also actually going through with) denying older people medical services solely because of their age. Indeed, it is regularly alleged that the fundamental imperatives of equal treatment and of equality among people are being violated (Knell 2017). Evidence suggests that when it comes to hospital beds there is an implicit rationalization influenced by age, to the detriment of victims (Schweda et al. 2018).

Even general medical and nursing care for older people is problematic. The more complicated an older person in need of care appears, the more susceptible they are to neglect and violence (Gordon and Brill 2001). This includes physical, sexual, and emotional abuse (Balsam and D’Augelli 2006). Just as with medical care for trans persons, there exists a tendency toward discrimination, insufficient care, and lack of research (Yarbrough 2018). Notably, it is thought that psychotherapeutic care for older people is sorely lacking (Elsässer et al. 2017). What is particularly striking is the fact that the older patient demographic is usually excluded from clinical studies, although the need for pharmaceuticals among this group is especially great. Consequently, there is less evidence of drug efficacy for this group (Schweda et al. 2018). With that said, research into aging is growing at a faster rate than trans health research (Schweda et al. 2018), although mostly in the sense of anti-aging rather than pro-aging. In any case, international journals such as *Aging Research*, *Mechanism of Aging and Development* and *Aging Cell* are documenting activity in this field.

The paradigm of individuality—according to which people plan their futures based on their own interests, needs, and preferences, doing so as independently as possible from other people and from gender (Schwiter 2011)—dictates that the autonomy of older people is emphasized as a central ethical principle in medical ethics. Even the WHO defines autonomy in the context of age as, most importantly, self-determination in everyday living: “Autonomy is the perceived ability to control, cope with and make personal decisions about how one lives on a day-to-day basis, according to one’s own rules and preferences” (World Health Organisation 2003). However, medical ethics is concerned with the problem of varying degrees of autonomy (Werren 2017). For example, the difference between autonomy of action and autonomy in decision-making, whereby the latter is also to be respected, is emphasized if autonomy of action is no longer possible due to multimorbidity that leads to impaired articulation. In this context, Advance Care Planning *(Vorausverfügung)* is discussed (Schweda et al. 2018).

## Transgender People in Older Age: Concept, Medicine, Ethics

Hardly any empirical research on aging in trans persons exists (Balsam and D’Augelli 2006; Bailey 2012; Newman and Price 2012). Trans persons remain invisible in the context of older people’s care and medicine (Carroll 2017; Stein 2012) because prejudices render older people’s facet of gender inconceivable, as if old people represent an amorphous, gray mass (Carroll 2017) whose gender and sexuality are merely “past glories”.

This lack of visibility is made even more problematic by the fact that the demographic of trans persons increasingly consists of older, geriatric people (Bailey 2012; Newman and Price 2012). This comes with new issues, one of which is of the medical sort: for example, the prominent issue of the long-term effectiveness of hormones, or their effects on the older body when taken in combination with other medications (Carroll 2017). However, social questions are being posed more frequently: for example, the extent to which transphobia and ageism go hand in hand and result in great physical strain. Until now, there has only been anecdotal evidence of this, but it is emerging that older people struggle to have their interests and preferences recognized even in an institutional context (Bailey 2012).

The statistical probability of trans persons’ making use of special medical or nursing help in old age is distinctly lower than for older cisgender people (Arthur 2015; Porter and Krinsky 2014). It is not rare for trans persons also to have no regular contact in older age with doctors (Carroll 2017). This corresponds to their general quality of health, similar to the demographic of trans persons in general; even in older age, cis people are in better health than their trans peers (Carroll 2017).

In older age, most trans and gender nonconforming people, similarly to cis people, express fewer growth targets than avoidance targets (Elsässer et al. 2017); although transitions are not just limited to younger years (Transgender Network Switzerland 2018) and a non-trivial number of people only undergo their gender reassignment in older age (Cook-Daniels 2006). This proves that there can still be significant growth goals in old age. A classic avoidance goal is being able to live alone. For older trans persons, this is accompanied by the desire to continue to be respected as a trans person and to be able to perform the practices associated with this. Since in older age limitations in the areas of physicality, social relationships, spatial behaviors, and mobility are increasingly present (Elsässer et al. 2017), these concerns are justified.

If trans and gender nonconforming people become multimorbid geriatric individuals—that is to say that they are suffering from pain, functional deficiencies (immobility, loss of bladder control, sensory impairments), increased vulnerability (likelihood of having a fall, complications due to multiple medications, etc.), as well as lower mental resilience which can be accompanied by fear and depression (Kuhlmey and Tesch-Römer 2013)—then they seem to share the same fate as very elderly cis people (Gangamma 2008). Despite the comparable medical situation of multimorbid patients, it must not be forgotten that old-aged trans persons also risk, alongside all the aforementioned losses, having their gender reassignment revoked in acute medical or paternalistic health and nursing care. For example, hormones could be discontinued because of possible interactions (Cook-Daniels 2006), or cosmetics could be ceased (Stein 2012).^14^ The probability of mental burden in trans persons rises sharply when their specific needs are no longer respected (Arthur 2015; Tinney et al. 2015). At the end of their lives, trans persons worry about whether or not they will be able to die as their own gender (Stein 2012; Witten 2014).^15^ All in all, depression and anxiety tend to be more widespread among elderly trans persons compared to their cis peers (Tinney et al. 2015).

## Spaces for Older Transgender People: A Contribution from Theological Ethics

Demands for places of visibility and spaces for the individual are regularly made in trans-positive studies: “[…] I attempt to narrate a decolonizing pedagogy of transing that might create space for trans-, for sovereignty, and for gender: what I think of as gender/sovereignty spaces” (Muñoz 2012, p. 23). Or with a particular focus on healthcare: “Very much at the heart of this book lies a call for health and social care practice […] to create the space for LGBT lives” (Ward 2012, p. 198); as well as: “Here, in the present, we have the opportunity to ensure that LGBT elders […] are included in all types of research studies and that there is room for them in all services provided” (Kimmel et al. 2006, p. 15). Such demands for spaces are also compounded by factual, non-metaphorical loss of space since transgender people have a higher chance of being violently attacked than cis people, therefore their social mobility is limited. Trans persons have a higher risk of being thrown out of their homes by their parents in their younger years, and of losing accommodation in their later years, leading to homelessness (Beemyn et al. 2012). Furthermore, the ethical focus in studies on trans persons includes metaphors for places and spaces whose exact ethical content often remains unclear, which will be examined here in more detail. In particular the focus lies on the question of whether older trans persons alone benefit from being allocated locations and spaces, or whether a nuanced concept of space allocation is essential.

Christian ethics are associated with concepts such as charity, loving your enemy, compassion (Herdt 2012), but also with prioritizing the individual above the universal; however, justice usually takes priority. In any case, the concrete meaning of this—that is to say, what exactly the consequences entail—is often left open (Wirth 2020). One implicit but also concretizing ethical formula in Hebrew and Christian traditions focuses on living environments, as found in concepts like brother–sisterhood, friendship, and Sabbath (Fischer 1998; Wirth 2019). Constitutively associated with this, though less recognized as such, is the notorious practice of offering a special space (Heß 2008). This rather general claim can be applied to the requirement of specific spaces for elderly trans persons and gender nonconforming individuals, as shall be outlined in this study. There are theological narratives within the Hebrew and Christian traditions that can foster space-awareness (Wirth 2018; Wirth 2020) when re-read in light of the problems elderly trans persons regularly face in Western cultures. Adonai, for instance, first appears in the Hebrew Bible as the creator of space in which other beings may exist; all in analogy with God’s own being, that as a trinity also implies a vast space for others (“spacious interior of the triune God”/“geräumiger Binnenraum des dreieinigen Gottes”) (Frettlöh 2001, p. 109). In this way, Adonai appears to be connected to the space created—so strongly that God also appears as a place (*maqom*) (Frettlöh 2001). This is not, however, in the sense that he occupies space; rather, God, by means of withdrawal or contraction, bestows space upon the vicinity (Frettlöh 2001). The New Testament stories, too, similarly to the Hebrew Bible, begin with narratives on space. First, the unparalleled presence of God for Christians appears in the birth of Jesus in the concrete space of Israel. Again, it is about God’s presence in a tangible place. Second, the first books of the New Testament begin with an existential search for space (Frettlöh 2001). This is how Jesus’s story begins: not with an aggressive taking up of space, but rather with withdrawing away from the occasionally inhospitable otherness of the other.

In Christian theology little thought has so far been given to whether a specifically spatial type of ethics summarizes the essential features of Judaism and Christianity, with practical potentials, and whether, if so, this could serve as a form of thought for a type of ethics that allows for trans-positive refigurations (Wirth 2019).^16^ A practical theological ethical format could benefit from this, characterized by the “affirmation of everyday life” or the “valorization of everyday life” (Fischer 2010, p. 35-36). Based on thinking about spatial offering, what Christianity defines as good is that which gives space to allow another to live, and therefore to be itself (Herdt 2009; Wirth 2020). “I am going there to prepare a place for you” (John 14:2) would then be not only an eschatological statement (Frettlöh 2001), but also an ethical one which elderly trans persons and gender nonconforming individuals, too, should benefit from.^17^

Such a theological ethics of space (Wirth 2020) might exhibit two components with regard to the specific vulnerability of elderly trans persons: on the one hand, a moment of withdrawal, contraction, retreat, without which there could be no room for the other. This also extends to God. In Hebrew theological tradition, on the other hand, such a withdrawal of God (*contraction Dei*) is considered a requirement for the existence of an other, a creation beside the absolute God. Contracting and offering space leaves behind a particular form of presence so as to avoid God’s complete absence, since making spaces available, only to then leave them and those who remain there, would be an ethically problematic format for spatial offering. A perceived presence can only exist through withdrawal. A sculpture by the artist Christoph Loos by the name of ZimZum shows this relationship very impressively; the name serves as a reminder of the idea rooted in the Kabbalah that God contracts for the benefit of creation (Wirth 2019). One main feature of the sculpture is long thin cones that point into the space with the bases of the cones facing the upper wall where they cast a light.^18^ The two ends of the cones can be interpreted as both presence and retreat, with the presence in the middle of the space taken back so that the emphasis is on the edge, thus leaving room for the other in the center; however, the place at the edge of the spatial offering is not insignificant since Loos arranges the cones to be bright and golden in their place of withdrawal to avoid creating a sense of absence. The sculpture is reminiscent of an antique four-poster bed, in reference to everyday life. The bed, much like a cocoon, acts as a metaphor for vulnerability and might be viewed in the context of this study as a place of both fear and help. Protection is also implied as a contraction in the context of this study, but only in the case that presence is not completely lost.

## Conclusion: The Ambivalent Maneuver of Withdrawal for the Sake of Elderly Trans Persons

There are good reasons for understanding Jewish and, *mutatis mutandis,* Christian ethics as ethics of giving space through contraction and withdrawal.^19^ Religious concepts of hospitality, friendship, Sabbath, and divine as well as human withdrawals, can be re-read in the lens of an ethics that is highly relevant to vulnerable groups affected by religious gender biases. Such an ethics stresses the outreach of such concepts for people in need of space. This also holds true for trans people, especially during the phase of being an older person. Since the normative status of actions of withdrawal for the sake of the other is much higher in comparison to the highly contested dogma of two essentialist sexes, figures of hospitality, friendship and self-withdrawal for the other find more ethical legitimation in a theological ethics perspective. The demand for space by elderly trans persons and gender nonconforming individuals in the health sectors within many Western countries finds itself confronted with proliferative prejudices about sex and gender. In those cases where these biases (un)consciously refer to (secularized) religious concepts of gender within the Hebrew and Christian traditions, references to the broader normative concept of withdrawal can help to foster a trans-positive environment.

The material-ethic subject matter of this study—the insufficient healthcare of older trans individuals and gender nonconforming people—besides introducing the relevance of such concepts for theological ethics, adds an amplification to the ethics of space and withdrawal in the context of the specific needs of elderly trans persons. Space giving through withdrawal marks the start of everything for trans and gender nonconforming persons (as for any other person). As a result, withdrawal of aggrandized bionomic and essentialist gender stereotypes is needed so that there are (specific) spaces for (elderly) trans persons. Spaces for trans persons and gender nonconforming individuals—spaces that assume a withdrawal or contraction by all those who have previously taken up trans spaces through ignorance, contempt, or violence—should not become spaces of absence. This is also important for older trans persons, who alone as seniors are often no longer able to update their wills, and who are dependent on places in which both strategies prevail: first contraction, then presence. In order to prevent the gaze of this presence of the other, the helper, from becoming a divesting gaze in the spaces of older trans persons, and to instead facilitate therapeutic and benevolent aid, the presence of the other in the space of contraction should be gentle and careful, finding its place not in the center, but on the edge. Ultimately, reflections on spaces and ethics, and on the relationship between contraction and presence in the care of much older trans persons, not only affect this particular group, but also contribute toward allowing all people to live freely and authentically (Girshick 2008; Singh and Bower 2018).

## Footnotes

1 Admittedly, this point of view initially puts a broad focus on the disadvantages of being trans and older (Kimmel et al. 2006). In effect, this is a result of more deficient prospects of medical care. The study comes down to naming the necessary circumstances for older trans persons to develop in a positive way, and in no way doubts that successful older trans individuals exist (Fabbre 2015).

2 The fact that the program from 21st January 2018 provoked enormous criticism and an official complaint from the Transgender Network Switzerland (TGNS) who deemed the relevant statements during the program as defamatory or inappropriate (see: www.tgns.ch/wp-content/uploads/2018-Beanstandung_Antwort-Ombudsstelle-SRF.pdf) exemplifies how necessary a constructive dialogue between theology and the trans community is.

3 The German original quote goes as follows: “Queer-Sein ist für mich eine gesellschaftspolitische Haltung. Manchmal bezeichne ich meine Geschlechtsidentität als trans, manchmal als queer und manchmal einfach als männlich. Ich bin einfach ich, Ivan.”

4 Cisgender refers to the Latin *cis*, meaning “on this side”, as an antonym to the *trans* prefix (Sigusch 1991).

5 However, the concept of identity also draws criticism since it does not capture the element of fluidity and development prevalent in the individual lives of trans persons (Rubin 2003). Yet trans persons differ here only in a gradual way, but not a categorical way, from cis people, who do not express monolithic identities either, and can, for example, also suppress their feelings and desires to a considerable degree.

6 Of course the process varies a lot between jurisdictions, and psychological assessment is not required everywhere (Aizura 2011).

7 In this context, trans persons experience diminished autonomy, since they themselves are only considered “primary experts on their own lives” to a limited extent (Shelley 2008, p. 3).

8 This is related to the ongoing pathologization of being trans as a mental disorder, although initial steps toward depathologization are being made (Reed et al. 2016; Tinney et al. 2015).

9 This is also applicable to trans children and trans adolescents, although they differ from the focus group of older trans persons and often use medical treatments successfully (Drescher and Byne 2013; Shires et al. 2017).

10 Particularly in the field of psychiatry, historical review shows proof of the guild of psychiatric physicians’ guardedness against trans persons who were regarded not only as mentally ill, but also as “willfully annoying” (Cook-Daniels 2006, p. 27).

11 Zowie Davy quotes Raymond, a trans-person: “I had a doctor who struck me off because he couldn’t treat something like me because he was a good Catholic” (2010, p. 108). Another trans person cites a different case where no specialized medical help was offered: “I don’t work on people like you, you’ll have to go to see your GP” (quoted in Shelley 2008, p. 65).

12 Citing the following references on the meaning of Gen 1:27 in context is also a concern of theological ethics with its scope of orientation through differentiation (Fischer 2010). The following speak against a trans-negative interpretation of Genesis: (1) In Gen 1:26, the image of God is spoken of. As always, what is understood from this is that gender can have no determining significance, because God is not portrayed as having a gendered body in the Hebrew Bible. God’s face and other body parts such as the nose, eye, ear, arm, and hand are alluded to. For this reason alone, a normative assignment of the genitals must be retracted. If, however, God’s gender-specific qualities are named, then it shows that both female and male aspects shape each of the respective metaphors (Maier 2014). (2) The gender categories named in Gen 1:27 are not understood as exclusive categories in the exegesis. On the contrary, it could be interpreted that the masculine and feminine are understood as two different sides within each individual (Frettlöh 2009). Even more widespread is the interpretation according to which the two sides exist within each individual as a sign of equality among different people. The categories of male and female can or must be understood in the sense of a common merism in Hebrew rhetoric. There are two opposing pairs, as well as young and old, big and small, for a whole field (Wirth 2015). (3) What is particularly decisive for the trans issue, occasionally invasive gender reassignment interventions, and the notorious criticism referencing Genesis, is the separation of the blessing given to the person from the blessing given to the descendants, as can be taken from Gen 1:28. Here, firstly and unconditionally, a blessing of the person is carried out; the task of reproduction is secondary to this (Wirth 2015). Despite the indisputable significance of raising a family, being human does not forcibly involve procreation, and God’s blessing is not contingent on genital activity.

13 This form of discrimination exists most notably in the fact that people can fall under the rubric of “old” irrespective of their actual situation, leaving them at a disadvantage. In this respect, it is “unjust discrimination” that is particularly objectionable (Knell 2017, p.133).

14 For trans persons who are clearly in older age, experiences of loss also mean being able to be mindful of what remains. In a context particularly crucial to trans persons, the body can gain new importance since, after all, it is the body that remains until the final breath. Despite all these losses, and although the older body provides enough gnawing torment, its loyalty and servitude can lead to a new respect, perhaps even tenderness (Améry 2005; Bozzaro 2014). Contempt toward the trans body from a third party, which despite all resistance also becomes a refuge for fantasies and transitions, stands out from an ethical perspective as particularly negative in this context.

15 However, there are also positive developments in this area. In US care facilities, the first support groups for older trans persons are being established in addition to those for gay and lesbian residents (Krinsky and Cahill 2017).

16 One prominent exception is Emmanuel Lévinas, who in his ethics explicitly spoke of a “radical turn” (Lévinas 1995, p. 181) in the face of the Other and also of the problem of usurpation, the act of taking up space to the detriment of the Other (Lévinas 1995).

17 However, one danger of models based on spatial offering lies in the discernible emphasis of the character of offering. This implies an authority who may decide to bestow this gift, although there is no obligation for them to do so. An argument must consequently be made for a colonialist misunderstanding of spatial ethics. In the context of trans issues, it should not give off the impression that trans persons should wait for the benevolence of their colonial rulers who do still remain colonial rulers, even if they wield the power to liberate (Shelley 2008). Rather, a circumstance with spatial ethics should be described that can also be described from a Christian perspective as a type of right, since space in which a safe life can be led would have never been allowed to be taken away from people.

18 https://christophloos.com/bildarchiv/skulpturen/1997-2000#&gid=1&pid=2 [accessed March 23, 2018].

19 Hans Jonas with his “ethics of self-withdrawal” (“Ethik der Selbstbeschränkung”) (2017, p. 320) has based the “principle of responsibility” in an technological age mainly on the Hebrew concept of withdrawal and *Zimzum* (Wirth 2019) and has also stressed the interconnection between Hebrew and Christian ethical thinking and its potential to comment on contemporary challenges (Jonas 2017; Müller 2008).
